# Using Machine Learning Methods to Predict Bone Metastases in Breast Infiltrating Ductal Carcinoma Patients

**DOI:** 10.3389/fpubh.2022.922510

**Published:** 2022-07-06

**Authors:** Wen-Cai Liu, Ming-Xuan Li, Shi-Nan Wu, Wei-Lai Tong, An-An Li, Bo-Lin Sun, Zhi-Li Liu, Jia-Ming Liu

**Affiliations:** ^1^Department of Orthopaedic Surgery, The First Affiliated Hospital of Nanchang University, Nanchang, China; ^2^Department of Clinical Medicine, The First Clinical Medical College of Nanchang University, Nanchang, China; ^3^Institute of Spine and Spinal Cord, Nanchang University, Nanchang, China

**Keywords:** breast cancer, infiltrating ductal carcinoma, bone metastases, machine learning, prediction

## Abstract

Breast cancer (BC) was the most common malignant tumor in women, and breast infiltrating ductal carcinoma (IDC) accounted for about 80% of all BC cases. BC patients who had bone metastases (BM) were more likely to have poor prognosis and bad quality of life, and earlier attention to patients at a high risk of BM was important. This study aimed to develop a predictive model based on machine learning to predict risk of BM in patients with IDC. Six different machine learning algorithms, including Logistic regression (LR), Naive Bayes classifiers (NBC), Decision tree (DT), Random Forest (RF), Gradient Boosting Machine (GBM), and Extreme gradient boosting (XGB), were used to build prediction models. The XGB model offered the best predictive performance among these 6 models in internal and external validation sets (AUC: 0.888, accuracy: 0.803, sensitivity: 0.801, and specificity: 0.837). Finally, an XGB model-based web predictor was developed to predict risk of BM in IDC patients, which may help physicians make personalized clinical decisions and treatment plans for IDC patients.

## Introduction

As one of the most common malignancies, breast cancer (BC) accounted for 30% of all cancers in women (1). The incidence of BC continued to increase at a rate of ~0.5% per year, which was attributed at least in part to the continued decline in fertility and increased body weight (2). In the cancer statistical report, the number of BC patients exceeded 2.1 million, and infiltrating ductal carcinoma (IDC) was the most common one among different types of BC (3, 4). Early diagnosis provides a favorable prognosis and better overall survival for BC patients. In North America, early screening for BC significantly increased the 5-year survival rate to over 80% for BC patients (5). In recent years, BC patients still had a high incidence of distant metastatic recurrence, which was an important indicator for poor prognosis. Recent studies have suggested that age, menopausal status, T, N stages, histological grade and HR/HER2 status were risk factors for BM in BC patients (6–8). For distant metastases, bone was the most common site, and more than 60% of BC patients developed bone metastases (BM) (9). Another study indicated over 20% of patients developed BM within an average follow-up duration of 8 years. And further survival prognostic modeling showed a 40-month median survival time for patients with BM (10). In addition, BC patients with BM often developed secondary clinical complexities that took up significant medical resources (11). Early diagnosis of BM from BC will help in the timely prevention and treatment of complications and increase the quality of life for BC patients.

Currently, precision medicine has preceded four concepts: predictive, personalized, preventive and participatory (12). The technology of big data analytics is becoming a clinical imperative (13). This means that we need to use advanced technology to analyze large amounts of medical data to provide recommendations for individualized treatment. Many studies have used machine learning (ML) techniques to study clinical risk factors associated with cancer metastases for early detection (14–16). It is known that the most common type of pathology in BC is IDC (4). But few studies focused on incorporating machine learning to predict the risk of BM from IDC patients.

In this study, we attempted to develop a predictive model to predict the BM risk in IDC patients based on machine learning, and to assist clinicians in implementing more rational clinical decisions as well as to enable patients to receive earlier treatment. Our contribution includes:

- Machine learning algorithms were used instead of traditional statistical regression methods to process data on clinical characteristics of IDC patients to identify patients at high risk for BM.
- This paper compared various algorithms that could be used to process patient data and identified XGB as the best method for processing the data. Further, the hyperparameters of the XGB algorithm were fine-tuned using a random search method to improve performance.
- We performed importance analysis of the features included in the model using the permutation importance method, and these features were further analyzed and understood from a clinical medicine perspective in the discussion section.
- This study proposed a machine learning based solution that could assist clinicians in making individualized diagnoses of BM for IDC patients.

The rest of the paper was organized as follows: the materials and methods section provided a detailed documentation of the materials and methods used, as well as the dataset description, statistical analyses, data pre-processing, feature engineering, classification algorithms used and evaluation metrics. Results of the experiment are discussed in Section Results and then further discussed in Section Discussion. Conclusions, limitations and future work section concluded the results and provided limitations and the future direction of the current work.

## Materials and Methods

### Study Population

**Figure 1:**
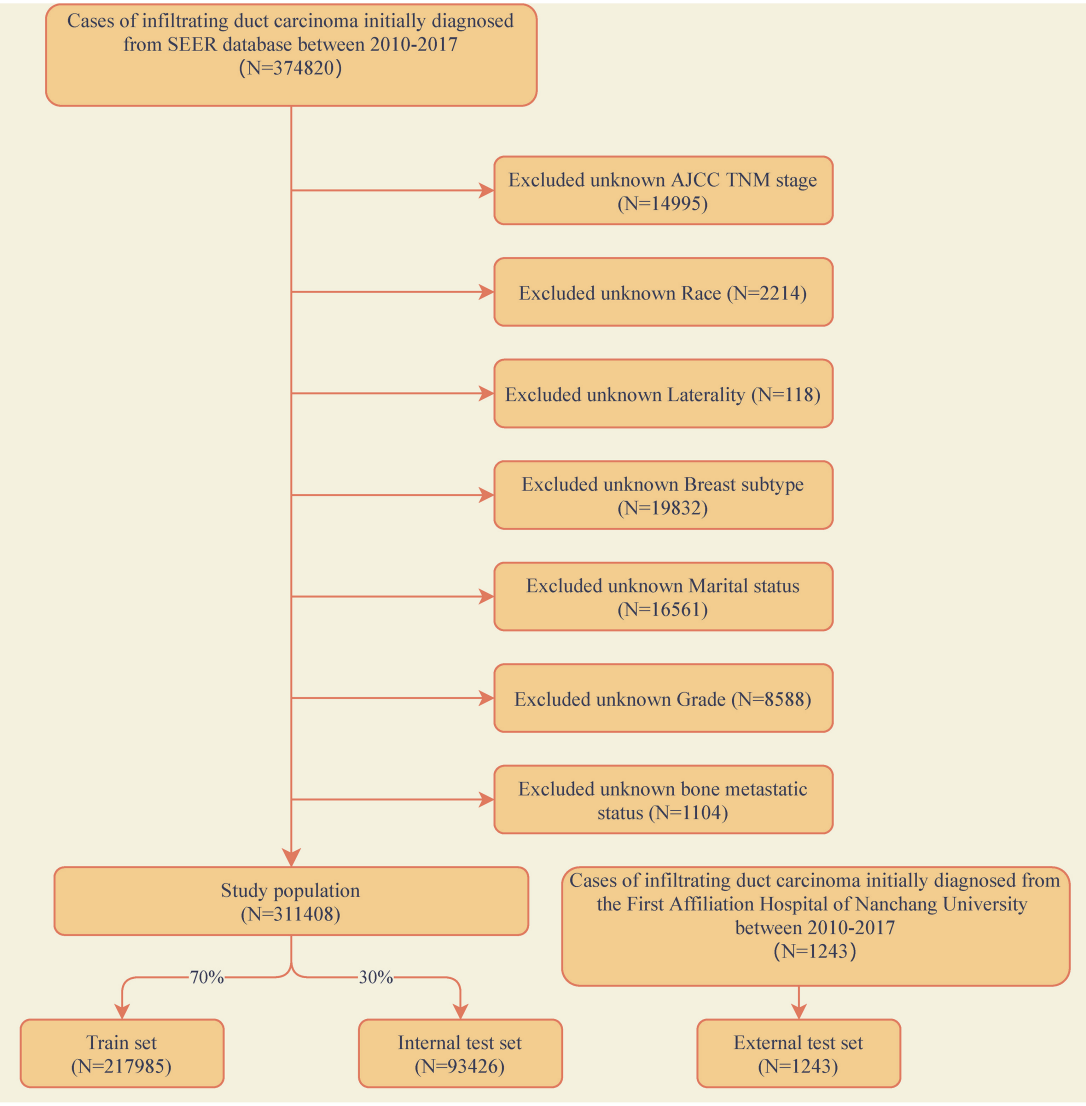
Flow diagram of the study population selected from the Surveillance, Epidemiology, and End Results (SEER) database and the First Affiliated Hospital of Nanchang University. According to the inclusion and exclusion criteria, a total of 311,408 patients of SEER were included in this study, and they were randomly cut into the training and internal test sets in a 7:3 ratio. Data from the First Affiliated Hospital of Nanchang University (*n* = 1,243) as an external test set.

The training and internal test set data was derived from the Surveillance, Epidemiology, and End Results (SEER) database, and the external test set data was derived from the First Affiliated Hospital of Nanchang University in China. A data set of 311,408 IDC patients was included from the SEER database (2010–2017) and sliced into a training set and an internal test set randomly in a ratio of 7:3. An external validation set included data from 1,243 IDC patients of our hospital (2010–2017). The exclusion criteria for clinical data were as follows: (1) unknown information of T, N stage, race, laterality, breast subtype, grade and marital status. (2) Other cases with unknown primary tumor and metastatic status. The detailed exclusion criteria were shown in Figure 1. Information of all variables was complete for these patients.

### Data Selection

We selected nine variables from the SEER database and our hospital that may affect BM in patients with IDC, including age at diagnosis, race, sex, grade, T, N stage, breast subtype, laterality and marital status. All cases included in this study were staged using the 7th edition of the AJCC TNM staging system and the relevant guidelines of the SEER project.

### Statistical Analyses

The statistical analyses in this study were all performed by Python (version 3.8, Python Software Foundation) and SPSS (version 26, IBM, USA). Data from SEER were randomly sliced into training and internal test sets in a ratio of 7:3 using python. The training set was used to construct the models, and the internal test set and external test set were used for model validation and evaluation. A heat map was drawn to determine the association among the variables. A univariate analysis was performed to compare variables between patients with and without BM. For categorical data, the chi-square test was used, and for continuous non-normally distributed data, the Wilcoxon rank-sum test was used. Variables with a *P* < 0.05 in univariate analysis were enclosed within the construction of machine learning models and multivariate logistic regression was performed to identify the risk factors of BM from IDC patients.

### Data Pre-processing and Feature Engineering

Category variables such as T and N stages were processed using label encoding methods. The univariate analysis was used to screen for meaningful combinations of features for predicting the risk of IDC patients with BM. Correlation analysis was used to analyze the correlation among the selected features. Feature importance analysis was performed on the variables based on the Permutation Importance principle (17, 18).

### Evaluation Metrics

The purpose of this study was to accurately predict the clinical outcome of a specific patient based on multiple variables. So the predictive power and accuracy of the model were important. Thus, to evaluate the model, we considered the area under the receiver operating characteristic curve (AUC), accuracy, sensitivity (recall rate) and specificity score in the study. The following terms were used in the equations: TP (True Positive); TN (True Negative); FP (False Positive); and FN (False Negative).


$$\begin{array}{l} Accuracy=\frac{TP+TN}{TP+TN+FP+FN} \end{array}$$

$$\begin{array}{l} sensitivity\;\left(recall\;rate\right)=\frac{TP}{TP+FN} \end{array}$$

$$\begin{array}{l} specificity=\frac{TN}{TN+FP} \end{array}$$

### Model Establishment

All the algorithmic models were built based on scikit-learn (version 0.24.2). The random oversampling method in imbalanced-learn (version 0.9.1) was used to deal with the imbalance of data distribution.

In this study, we used six different machine learning algorithms: Logistic regression (LR), Naive Bayes classifiers (NBC), Decision tree (DT), Random Forest (RF), Gradient Boosting Machine (GBM) and Extreme gradient boosting (XGB) (19–24). The ML algorithms were trained and adjusted to predict the BM in IDC patients. Random search method in scikit-learn was used to adjust the hyperparameters of the model. Then, the predictive performance of the ML models was evaluated in internal 10-fold cross-validation of the train set, internal and external test sets and the AUC, accuracy, sensitivity (recall rate) and specificity score were evaluated. Then, we selected the best-performing model to build a web predictor.

## Results

### Demographic Baseline Characteristics

A total of 311,408 cases who were first diagnosed with IDC in the SEER database from 2010 to 2017 were included. Of these cases, 7,949 (2.55%) complicated with BM and 303,459 (97.45%) without BM. All demographic and clinicopathological characteristics of these patients were demonstrated in detail in Table 1. All cases were randomly divided into a training set (*n* = 217,985) and an internal test set (*n* = 93,426) in a ratio of 7:3. Data for the external test set were derived from IDC patients firstly diagnosed in our hospital from 2010 to 2017 (*N* = 1,243). The details of the training and test sets were shown in Table 2.

**Table 1:** Clinical and pathological characteristics of study population.

**Variables**	**ALL**	**NBM**	**BM**
	***N* = 311,408**	***N* = 303,634**	***N* = 7,774**
**Age**			
<50	65,967 (21.2%)	64,129 (21.1%)	1,838 (23.6%)
≥50	245,441 (78.8%)	239,505 (78.9%)	5,936 (76.4%)
**Sex**			
Female	308,805 (99.2%)	301,162 (99.2%)	7,643 (98.3%)
Male	2,603 (0.8%)	2,472 (0.8%)	131 (1.7%)
**Race**			
American Indian/Alaska Native	1,848 (0.6%)	1,809 (0.6%)	39 (0.5%)
Asian or Pacific Islander	28,929 (9.3%)	28,312 (9.3%)	617 (7.9%)
Black	35,011 (11.2%)	33,749 (11.1%)	1,262 (16.2%)
White	245,620 (78.9%)	239,764 (79.0%)	5,856 (75.3%)
**Grade**			
Grade I (well differentiated)	65,791 (21.1%)	65,252 (21.5%)	539 (6.9%)
Grade II (moderately differentiated)	132,463 (42.5%)	128,913 (42.5%)	3,550 (45.7%)
Grade III (poorly differentiated)	112,515 (36.1%)	108,858 (35.9%)	3,657 (47.0%)
Grade IV (undifferentiated)	639 (0.2%)	611 (0.2%)	28 (0.4%)
**Breast subtype**			
HR-/HER2- (triple negative)	38,740 (12.4%)	37,927 (12.5%)	813 (10.5%)
HR-/HER2+ (HER2 enriched)	15,803 (5.1%)	15,246 (5.0%)	557 (7.2%)
HR+/HER2- (Luminal A)	219,700 (70.6%)	214,764 (70.7%)	4,936 (63.5%)
HR+/HER2+ (Luminal B)	37,165 (11.9%)	35,697 (11.8%)	1,468 (18.9%)
**T stage**			
T1	191,204 (61.4%)	190,153 (62.6%)	1,051 (13.5%)
T2	93,067 (29.9%)	90,289 (29.7%)	2,778 (35.7%)
T3	15,307 (4.9%)	14,031 (4.6%)	1,276 (16.4%)
T4	11,830 (3.8%)	9,161 (3.0%)	2,669 (34.3%)
**N stage**			
N0	215,120 (69.1%)	213,308 (70.3%)	1,812 (23.3%)
N1	72,080 (23.1%)	68,326 (22.5%)	3,754 (48.3%)
N2	15,459 (5.0%)	14,400 (4.7%)	1,059 (13.6%)
N3	8,749 (2.8%)	7,600 (2.5%)	1,149 (14.8%)
**Laterality**			
Left	157,489 (50.6%)	153,472 (50.5%)	4,017 (51.7%)
Right	153,919 (49.4%)	150,162 (49.5%)	3,757 (48.3%)
**Marital status**			
Married	261,707 (84.0%)	255,829 (84.3%)	5,878 (75.6%)
Unmarried	49,701 (16.0%)	47,805 (15.7%)	1,896 (24.4%)

*BM, Bone metastasis; NBM, No bone metastasis; HR, hormone receptor; HER2, human epidermal growth factor receptor 2*.

**Table 2:** Clinical and pathological characteristics of training set and test set.

**Variables**	**Training set**		**Internal test set**		**External test set**	
	**NBM (%) (*n* = 212,567)**	**BM (%) (*n* = 5,418)**	**NBM (%) (*n* = 91,067)**	**BM (%) (*n* = 2,356)**	**NBM (%) (*n* =1,068)**	**BM (%) (*n* = 175)**
**Age**						
<50	45,017 (21.2)	1,270 (23.2)	19,112 (21.0)	568 (24.1)	358 (33.5)	69 (39.4)
≥50	167,550 (78.8)	4,148 (76.8)	71,955 (79.0)	1,788 (75.9)	710 (66.5)	106 (60.6)
**Sex**						
Female	210,814 (99.2)	5,322 (98.2)	90,348 (99.2)	2,321 (98.5)	1,064 (99.6)	173 (98.9)
Male	1,753 (0.8)	96 (1.8)	719 (0.8)	35 (1.5)	4 (0.4)	2 (1.1)
**Race**						
American Indian/Alaska Native	1,267 (0.6)	24 (0.4)	542 (0.6)	15 (0.6)	0 (0.0)	0 (0.0)
Asian or Pacific Islander	19,981 (9.4)	415 (7.7)	8,331 (9.1)	202 (8.6)	1,068 (100.0)	175 (100.0)
Black	23,645 (11.1)	888 (16.4)	10,104 (11.1)	374 (15.9)	0 (0.0)	0 (0.0)
White	167,674 (78.9)	4,091 (75.5)	72,090 (79.2)	1,765 (74.9)	0 (0.0)	0 (0.0)
**Grade**						
Grade I (well differentiated)	45,558 (21.4)	385 (7.1)	19,694 (21.6)	154 (6.5)	202 (18.9)	11 (6.3)
Grade II (moderately differentiated)	90,360 (42.5)	2,461 (45.4)	38,553 (32,631)	1,090 (46.3)	494 (46.3)	82 (46.9)
Grade III (poorly differentiated)	76,227 (35.9)	2,555 (47.2)	32,631 (35.8)	1,102 (46.8)	369 (34.6)	81 (46.3)
Grade IV (undifferentiated)	422 (0.2)	18 (0.3)	189 (0.2)	10 (0.4)	3 (0.3)	1 (0.6)
**Breast subtype**						
HR-/HER2- (triple negative)	26,586 (12.5)	569 (10.5)	11,341 (12.5)	244 (10.4)	95 (8.9)	9 (5.1)
HR-/HER2+ (HER2 enriched)	10,737 (5.1)	377 (7.0)	4,509 (5.0)	180 (7.6)	73 (6.8)	16 (9.1)
HR+/HER2- (Luminal A)	150,391 (70.7)	3,453 (63.7)	64,373 (70.7)	1,483 (62.9)	765 (71.6)	118 (67.4)
HR+/HER2+ (Luminal B)	24,853 (11.7)	1,019 (18.8)	10,844 (11.9)	449 (19.1)	135 (12.6)	32 (18.3)
**T stage**						
T1	132,936 (62.5)	750 (13.8)	57,217 (62.8)	301 (12.8)	639 (59.8)	12 (6.9)
T2	63,445 (29.8)	1,929 (35.6)	26,844 (29.5)	849 (36.0)	350 (32.8)	62 (35.4)
T3	9,829 (4.6)	866 (16.0)	4,202 (4.6)	410 (17.4)	52 (4.9)	33 (18.9)
T4	6,357 (3.0)	1,873 (34.6)	2,804 (3.1)	796 (33.8)	27 (2.5)	68 (38.9)
**N stage**						
N0	149,389 (70.3)	1,261 (23.3)	63,919 (70.2)	551 (23.4)	746 (69.9	41 (23.4)
N1	47,801 (22.5)	2,630 (48.5)	20,525 (22.5)	1,124 (47.7)	241 (22.6)	79 (45.1)
N2	10,019 (4.7)	722 (13.3)	4,381 (4.8)	337 (14.3)	60 (5.6)	30 (17.1)
N3	5,358 (2.5)	805 (14.9)	2,242 (2.5)	344 (14.6)	21 (2.0)	25 (14.3)
**Laterality**						
Left	107,409 (50.5)	2,763 (51.0)	46,063 (50.6)	1,254 (53.2)	523 (49.0)	95 (54.3)
Right	105,158 (49.5)	2,655 (49.0)	45,004 (49.4)	1,102 (46.8)	545 (51.0)	80 (45.7)
**Marital status**						
Married	179,137 (84.3)	4,079 (75.3)	76,692 (84.2)	1,799 (76.4)	915 (85.7)	133 (76.0)
Unmarried	33,430 (15.7)	1,339 (24.7)	14,375 (15.8)	557 (23.6)	153 (14.3)	42 (24.0)

*BM, Bone metastasis; NBM, No bone metastasis; HR, hormone receptor; HER2, human epidermal growth factor receptor 2*.

### Univariate Analysis and Multivariate Logistic Regression Analysis

According to the univariate analysis, patients' age, gender, race, grade, T, N stage, breast subtype and marital status were significantly associated with BM in patients with IDC (*P* < 0.05; Table 3). Variables with a *P* < 0.05 in the univariate analysis were selected for multivariate logistic regression analysis, in order to identify the risk factors of BM in IDC patients. According to these results, age, race, grade, T, N stage, breast subtype, and marital status were found to be independent factors for BM (Table 3).

**Table 3:** Univariate analysis and multivariate logistic regression analysis of variables.

**Variables**	**Univariate analysis**		**Multivariate logistic regression analysis**	
	**Chi-Square**	***P-*value**	**OR (95%CI)**	***P-*value**
**Age**	12.612	<0.001		
<50			Reference	
≥50			1.122 (1.053–1.196)	<0.001^*^
**Sex**	56.360	<0.001		
Female			Reference	
Male			1.185 (0.946–1.485)	0.140
**Race**	157.012	<0.001		
American Indian/Alaska Native			Reference	
Asian or Pacific Islander			1.179 (0.764–1.818)	0.456
Black			1.641 (1.071–2.516)	0.023^*^
White			1.550 (1.016–2.365)	0.042^*^
**Grade**	716.162	<0.001		
Grade I (well differentiated)			Reference	
Grade II (moderately differentiated)			1.546 (1.380–1.732)	<0.001^*^
Grade III (poorly differentiated)			1.201 (1.066–1.353)	0.003^*^
Grade IV (undifferentiated)			1.314 (0.786–2.197)	0.298
**Breast_subtype**	317.014	<0.001		
HR-/HER2- (triple negative)			Reference	
HR-/HER2+ (HER2 enriched)			1.314 (1.143–1.510)	<0.001^*^
HR+/HER2- (Luminal A)			1.767 (1.600–1.951)	<0.001^*^
HR+/HER2+ (Luminal B)			2.003 (1.793–2.239)	<0.001^*^
**T stage**	17,446.197	<0.001		
T1			Reference	
T2			3.379 (3.464–4.149)	<0.001^*^
T3			8.810 (7.896–9.830)	<0.001^*^
T4			27.233 (24.635–30.104)	<0.001^*^
**N stage**	6,878.970	<0.001		
N0			Reference	
N1			3.061 (2.843–3.295)	<0.001^*^
N2			2.645 (2.387–2.932)	<0.001^*^
N3			4.390 (3.953–4.875)	<0.001^*^
**Laterality**	0.461	0.497		
Left			-	-
Right			-	-
**Marital status**	318.307	<0.001		
Married			Reference	
Unmarried			1.363 (1.272–1.460)	<0.001^*^

*BM, Bone metastasis; NBM, No bone metastasis; HR, hormone receptor; HER2, human epidermal growth factor receptor 2*.

* *P < 0.05*.

### Correlation Analysis of Features

**Figure 2:**
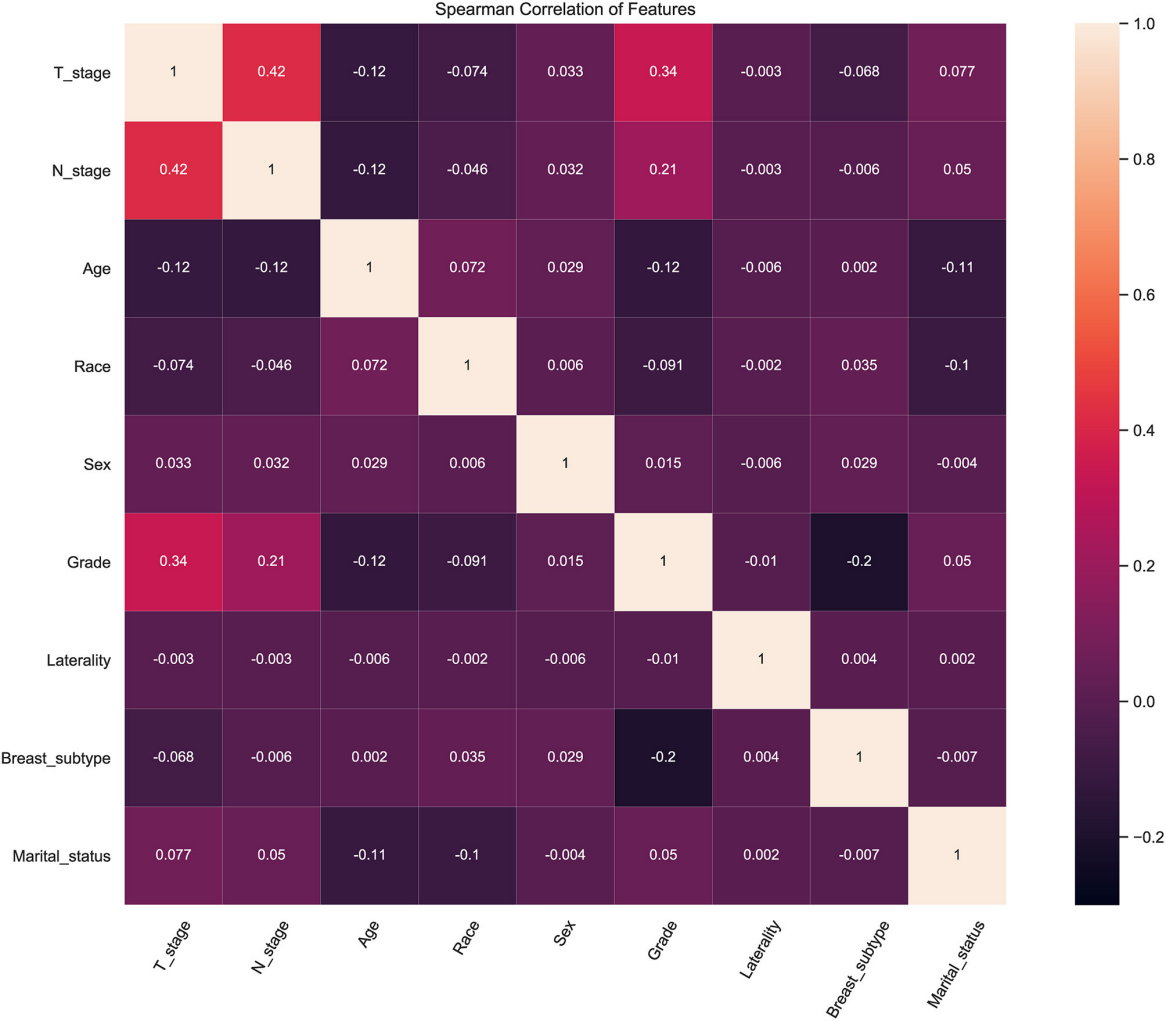
Results of correlation analysis between all variables.

To identify the effect of these features on prediction, correlation tests between each other were performed. Correlation analysis among dataset features provides information about the degree of interaction among features. The heat map showed the relevance of these features on the prediction of ML algorithm (Figure 2). The figure showed a positive correlation among T-stage, N-stage and pathological grade. This was consistent with clinical experience that poorly differentiated tumor tissue was poorly demarcated from surrounding normal tissue, which meant it was more aggressive.

### Relative Feature Importance on Prediction

**Figure 3:**
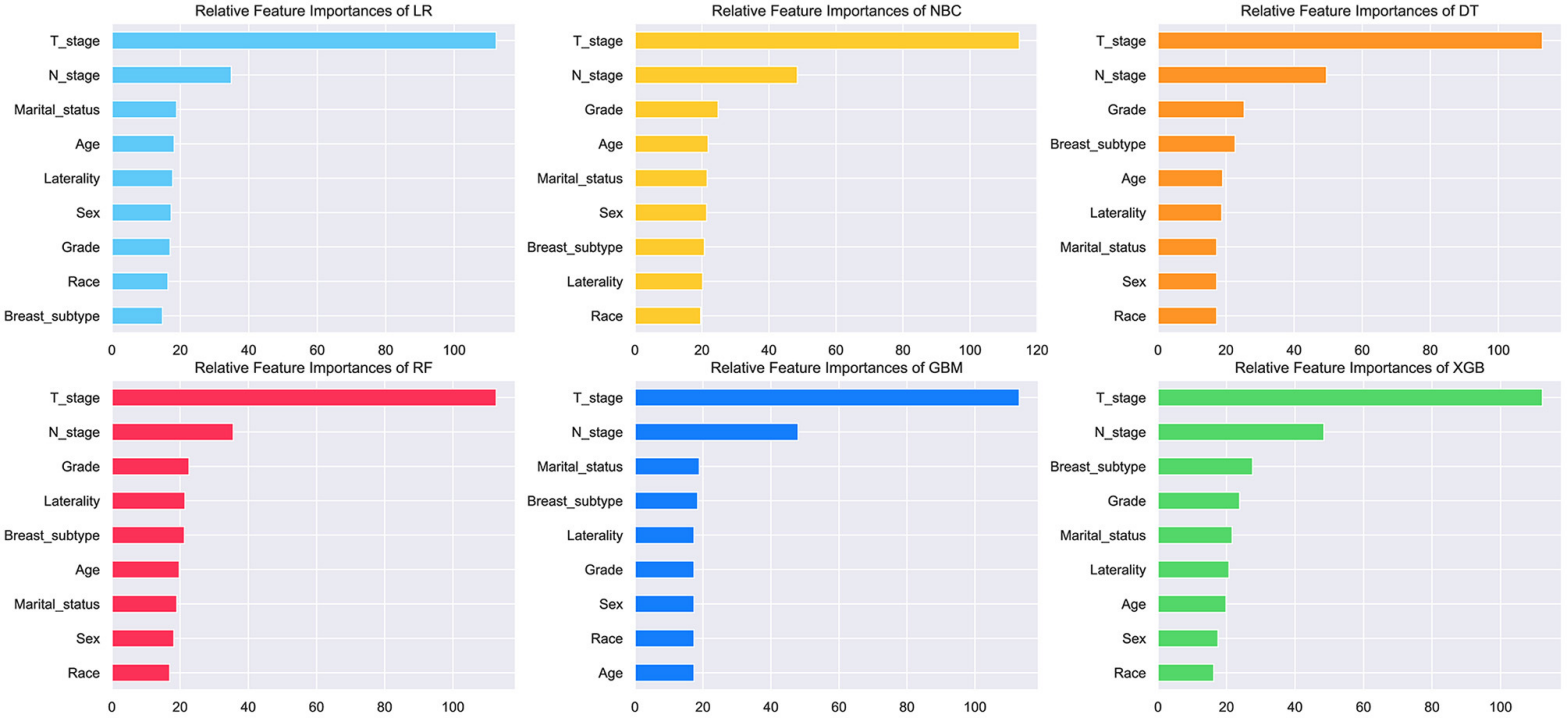
Relative feature importance of different models. The plot showed the ranking of the relative importance of features in all models.

The feature importance of each ML model for predicting BM was illustrated in Figure 3. Permutation importance principle was used to analyze the relative feature importance on the variables in each ML model. The basic idea of the principle was: (1) Train the model. (2) Disrupt the data in one of the columns and use that dataset for prediction, evaluating the decrease in prediction accuracy to reflect the importance of that feature variable. (3) Restore the validation dataset and repeat the second step to analyze the other feature variables. Although the relative feature importance in different ML models varied slightly, T, N stage, breast subtype, grade, and marital status were the top-ranked variables in most models. In contrast, the race was the last one in most models. But it also contributed to the model. In the XGB model, the relative feature importance was sorted in descending order by T, N stage, breast subtype, grade, marital status, laterality, age, sex, and race.

### Model Performance

**Figure 4:**
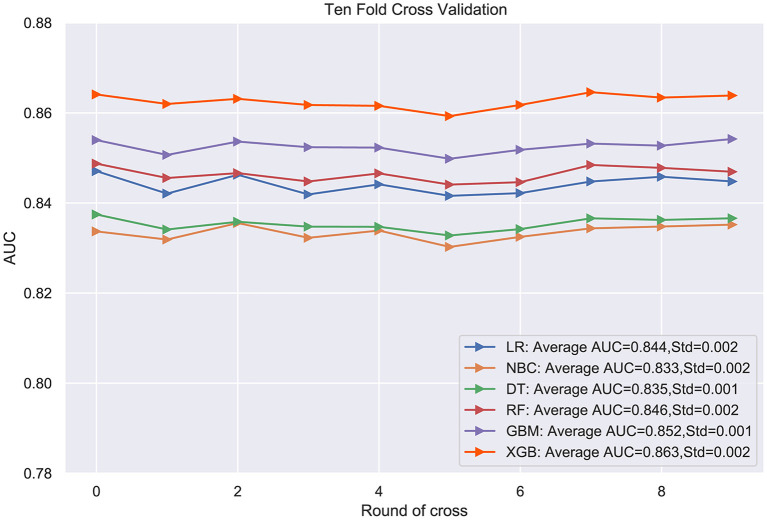
Ten-fold cross-validation results of different machine learning models in the training set. DT, Decision tree; LR, Logistic regression; GBM, Gradient Boosting Machine; NBC, Naive Bayes classification; RF, Random Forest; XGB, Extreme gradient boosting.

**Figure 5:**
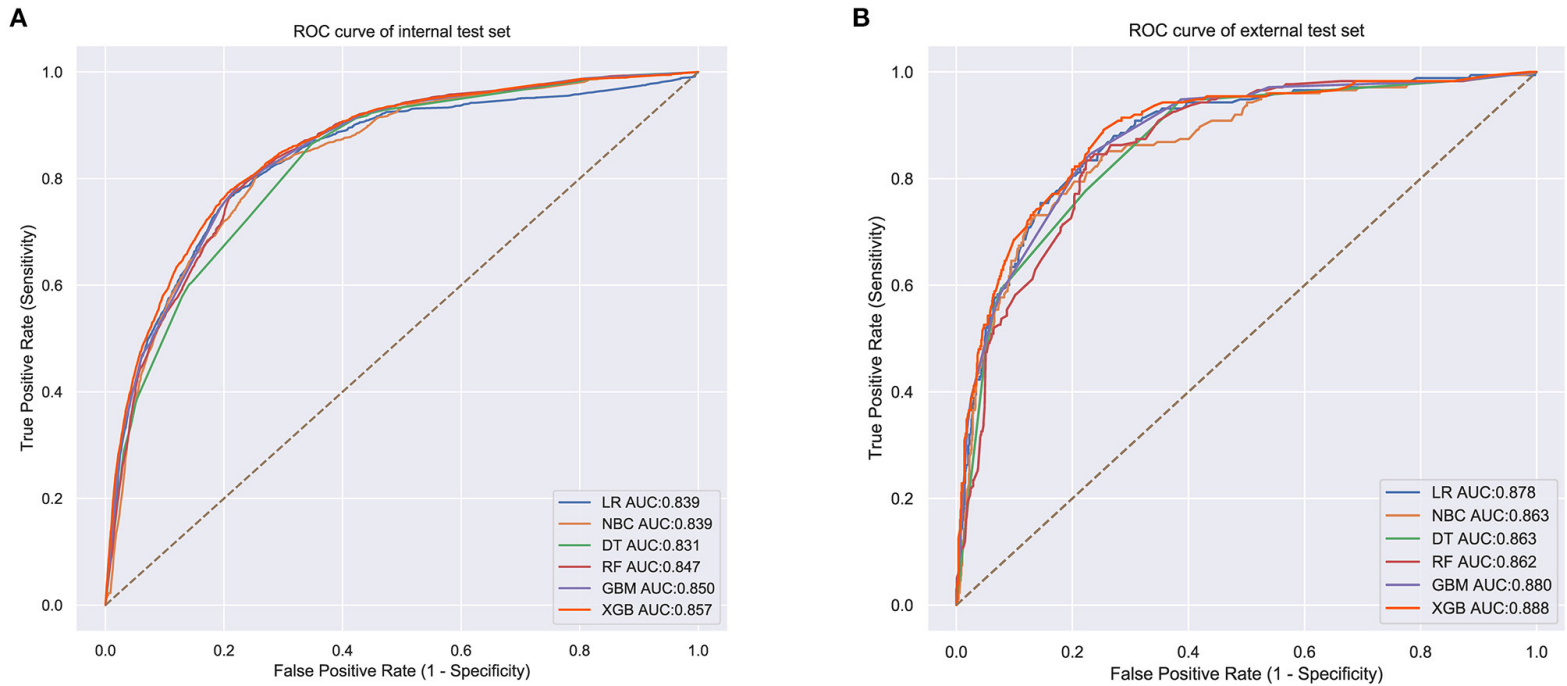
**(A)** Internal test ROC curve of different machine learning models. **(B)** External test ROC curve of different machine learning models.

**Figure 6:**
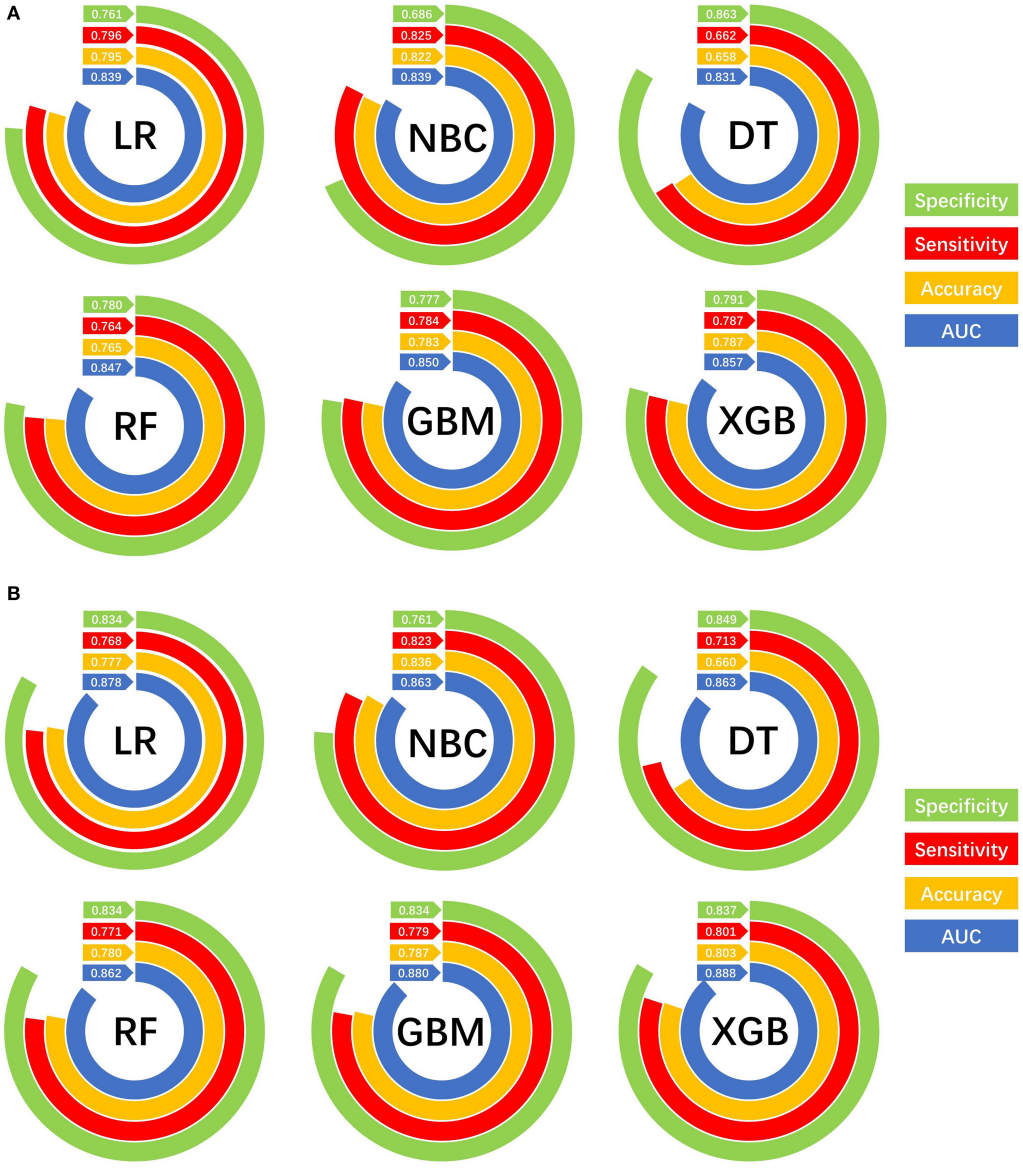
Prediction performances of different machine learning models. **(A)** Internal validation of different machine learning models. **(B)** External validation of different machine learning models.

The prediction performance of all models was compared with the internal 10-fold cross-validation of the training set, internal and external test sets, as shown in Figures 4–6 and Table 4. In the internal test set, the XGB model was the best performer with an AUC of 0.857, an accuracy of 0.787, a sensitivity of 0.787 and a specificity of 0.791. In the external test set, the XGB model showed a relatively better performance with an AUC of 0.888, an accuracy of 0.803, a sensitivity of 0.801 and a specificity of 0.837 (Table 4). Finally, we used the highest-performing model to create a web predictor.

**Table 4:** Comparison of prediction performances among different models for bone metastasis.

**Models**	**Internal test**				**External test**			
	**AUC**	**Accuracy**	**Sensitivity (recall rate)**	**Specificity**	**AUC**	**Accuracy**	**Sensitivity (recall rate)**	**Specificity**
LR	0.839	0.795	0.796	0.761	0.878	0.777	0.768	0.834
NBC	0.839	0.822	0.825	0.686	0.863	0.836	0.823	0.761
DT	0.831	0.658	0.662	0.836	0.863	0.660	0.713	0.849
RF	0.847	0.765	0.764	0.780	0.862	0.780	0.771	0.834
GBM	0.850	0.783	0.784	0.777	0.880	0.787	0.779	0.834
XGB	0.857	0.787	0.787	0.791	0.888	0.803	0.801	0.837

*DT, Decision tree; LR, Logistic regression; GBM, Gradient Boosting Machine; NBC, Naive Bayes classification; RF, Random Forest; XGB, Extreme gradient boosting*.

### Web Predictor

**Figure 7:**
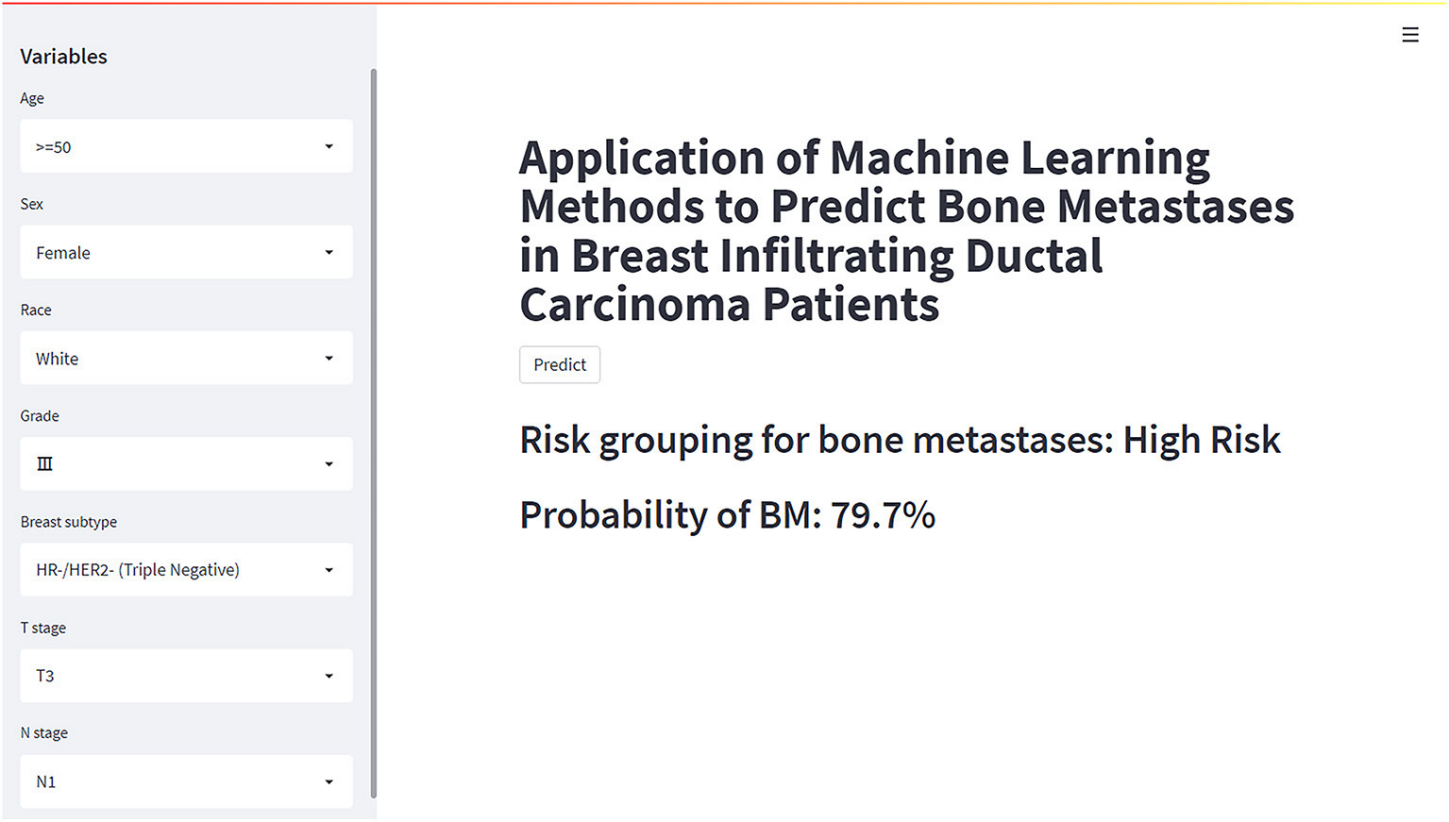
The web calculator for predicting bone metastases in breast infiltrating duct carcinoma patients.

Based on the XGB model, a ML algorithm for optimal predictive performance, the web predictor mentioned above was developed to predict the risk of BM in IDC patients. We can predict the BM risk in IDC patients simply by setting variables in the sidebar of the website (Figure 7) (https://share.streamlit.io/liuwencaincu/breast-cancer/main/breast.py).

## Discussion

Breast cancer was the most common malignant tumor in women, and IDC accounted for about 80% of all BC cases (3). BC patients who had BM were more likely to have poor prognosis and bad quality of life (25), and earlier attention to patients at a high risk of BM was important. However, metastatic BC was a highly heterogeneous disease, and bone was the most common site of distance metastasis from BC (3, 5, 26). A decline in quality of life was common among patients with BC who suffered from BM. And the risk of skeletal-related events significantly increased following BM in BC patients, such as pain, fracture and hypercalcemia (27, 28). The life expectancy of patients with BC benefitted from early detection and systemic therapy. Therefore, the prognosis of patients with late detection was often poor against a background of increasing incidence of BM.

Bone scintigraphy is often used to distinguish BC patients likely to develop BM. However, this method may not be suitable for early screening due to the expense and radiation damage associated with bone imaging. To help address these potential problems, we built a predictive model using ML technologies to predict BM in IDC patients and identify patients at a high risk of BM.

Presently, new techniques in ML and artificial intelligence (AI) help us succeed in translational research in many fields (29–31). Given the success of ML techniques in other fields, coupled with the large amount of data available in healthcare, ML techniques will have a promising future in the medical field (32–34). In particular, the emergence of electronic medical records (EMR) has generated a large accumulation of clinical data sets, including clinical diagnoses and laboratory data. ML in the medical field can lead to accurate diagnosis and personalized patient care (35). Since the outbreak of the COVID-19, scientists have used ML techniques to develop a variety of predictive and diagnostic models based on big clinical data from patients to help health care professionals work together to address the pandemic (36–38). Statistical and comprehensive reviews of ML in medical diagnosis by Bhavsar et al. (35, 39) suggested that ML techniques can help medical professionals reduce diagnostic errors, improve healthcare services, and cut treatment costs. And in the cancer metastases field, some clinical prediction models in predicting the risk of BM based on ML algorithms have been developed to assist clinicians in personalizing patient diagnosis (40, 41).

This study was novel in using ML algorithms to predict the risk of BM in IDC patients. To our knowledge, researchers in the medical field have only used traditional linear statistical models to predict the risk of BM in IDC patients, and few studies applied ML techniques to them (42). A ML model based on XGB algorithm was developed to accurately predict the risk of BM, outperforming other models developed in this study. The model established in this study had great discrimination and showed satisfactory specificity and sensitivity.

In this study, several models were constructed and validated to predict BM risk in patients with IDC using current ML methods, and logistic regression analysis demonstrated that age, race, grade, T, N stage, breast subtype, and marital status were independent risk factors for BM. After analyzing the performance of six ML algorithms, we found that the XGB method performed the best (AUC-0.888). The XGB algorithm added a regular term to the objective function to control the complexity of the model and avoid overfitting, while supporting column sampling to enhance the stability of the model (19). This may be part of the reason why it achieved the best performance in our study. To increase the practicality of this model implementation, we created an online web calculator for calculating individual BM probability in IDC patients.

Previous study indicated that age and race were investigated broadly as risk factors for BC metastasis (43). It was reported that white women were less likely to have BM from BC than black women (44). In our study, blacks also had a higher incidence of BM than those of Whites, American Indians and Asia-Pacific people. Chen et al. and Wang et al. found that elderly women were more likely to develop BM from BC (45, 46). In the present study, we found that advanced age was a risk factor for BM in patients with IDC.

Tumor size was positively correlated with the chance of BM. Yazdani et al. found that tumor size was a risk factor of BM from BC and a larger tumor size increased the likelihood of BM (47). In this study, patients with N3 stage were significantly more likely to develop BM than patients with other stages. And the average N stage in patients with BM was higher than that of the non-metastatic population. Yazdani et al. reported that more cancerous axillary lymph nodes increase the risk of BM (47). Colleoni et al. also found that there was the highest frequency of BM in patients who had four or more cancerous axillary lymph nodes (48). In addition, patients with higher-grade IDC had a higher risk of BM than those with grade I (Well differentiated) in this study. One reason for this may be that poorly differentiated tumor tissue was poorly demarcated from surrounding normal tissue, which meant it was more aggressive.

Additionally, it has been found that different breast subtypes showed different trends of BM. Previous study indicated that the incidence of BM was higher in patients with HR+/HER2- (Luminal A) and HR+/HER2+ (Luminal B) (49). In the current study, we found patients with HR+/HER2+ (Luminal B) were more likely to develop BM than those with other subtypes of BC.

Another risk factor for BM in our model was marital status. Unmarried patients had a higher risk of BM than those who were married. Zhao et al. and Gao et al. reported that marital status was a prognostic factor influencing the survival of metastatic BC patients (50, 51). Thus, we believed that the lifestyle habits and psychological factors of unmarried women may influence the distance metastasis of IDC patients.

As far as we know, this study was the first attempt to use ML algorithms to predict the risk of BM for IDC patients. Although some previous prediction models for BC based on the SEER database have been developed, it verified just from the SEER database and whether it can be used in different regions was not clear (26, 45). In addition, these studies only used nomogram as a visualization tool and did not provide a web predictor. External test of the model was important to validate the stability of the model for different regional populations. Therefore, in this study, we used data from the SEER database to build a prediction model based on the XGB algorithm and validated it with a cohort from China. Through our predictive model, we can predict the risk of BM in patients with IDC in the early stage, indicate the related risks before the progression to the late stage, and accept the corresponding treatment regimen as soon as possible, which can significantly improve the prognosis of IDC patients. Furthermore, we developed a web predictor based on the model to predict the risk of BM in IDC patients. Clinicians can easily enter information about the patient's relevant variables into the model on the web page, and the model will calculate the patient's risk of developing BM. Nowadays, precision medicine has preceded four concepts: predictive, personalized, preventive and participatory. Due to the ML model we built, we can predict the risk of BM to a particular patient. The rapidity and accuracy of the prediction output allowed clinicians to make personalized decisions for their patients and could be used as a basis for clinicians to explain their decisions to patients and involved them in their treatment choices. From the clinician's point of view, substantial advances in ML had potential implications in clinical practice, including diagnosis, risk stratification and prognosis, treatment planning, and advances in precision medical methods (52). Of course, they always had the final decision when it came to interpretation based on their domain expertise.

## Conclusions, Limitations, and Future Work

Overall, this study used ML algorithms to construct and validate a clinical prediction model for predicting the risk of BM in patients with IDC based on large samples. Among all these algorithms, XGB performed the best. And we built an easy-to-use web calculator based on the XGB model, which can help physicians to individualize the diagnosis and treatment of BM in IDC patients.

Although our model achieved good results in prediction, there were still some limitations in it. First, it was a retrospective study, which needed to be further verified by prospective study. Second, only one external validation set was used to validate the model, and further efforts were required to validate the performance of the model in a more diverse population. Third, the SEER database just recorded the initial diagnosis of a patient, which meant that further information was lack and we were unable to access this information for further analysis.

For future work, we will focus on prospective and diverse population validation of the models to verify the performance and stability. These models are then expected to be integrated into applications that assist clinicians in medical decision-making. This can be a step toward a semi-autonomous diagnostic system that can assist clinicians in making individualized diagnoses of BM for IDC patients.

## Data Availability Statement

The datasets presented in this study can be found in online repositories. The names of the repository/repositories and accession number(s) can be found below: https://seer.cancer.gov/.

## Ethics Statement

We received permission to access the research data file in the SEER program from the National Cancer Institute, US. Approval was waived by the Local Ethics Committee, as SEER data is publicly available and de-identified. This study was approved by the Ethics Committee of the First Affiliated Hospital of Nanchang University, and cases from the First Affiliated Hospital of Nanchang University signed a written informed consent form.

## Author Contributions

W-CL and J-ML designed the study. W-CL, S-NW, W-LT, A-AL, and B-LS performed analysis and generated the figures and tables. W-CL, M-XL, and S-NW wrote the manuscript. Z-LL and J-ML critically reviewed the manuscript. All authors have read and approved the manuscript.

## Funding

This work was supported by the Department of Science and Technology Program of Jiangxi Province, China (No. 20203BBG73045).

## Conflict of Interest

The authors declare that the research was conducted in the absence of any commercial or financial relationships that could be construed as a potential conflict of interest.

## Publisher's Note

All claims expressed in this article are solely those of the authors and do not necessarily represent those of their affiliated organizations, or those of the publisher, the editors and the reviewers. Any product that may be evaluated in this article, or claim that may be made by its manufacturer, is not guaranteed or endorsed by the publisher.
